# Hoffmann’s syndrome necessitating forearm fasciotomy: a case report

**DOI:** 10.1186/s13256-020-2351-x

**Published:** 2020-03-02

**Authors:** Erling Aarsæther, Ragnar Joakimsen, Hanne Halvorsen, Trude Sildnes, Olav Sivertsen, Jan Due

**Affiliations:** 1 Department of Urology and Endocrine Surgery, University Hospital of North Norway, Tromsø, Norway; 2 Department of Clinical Medicine, The Arctic University of Norway, Tromsø, Norway; 3 Department of Endocrinology, University Hospital of North Norway, Tromsø, Norway; 4 Department of Clinical Pathology, University Hospital of North Norway, Tromsø, Norway; 5 Department of Radiology, University Hospital of North Norway, Tromsø, Norway; 6 Department of Anesthesia, University Hospital of North Norway, Tromsø, Norway

**Keywords:** Compartment syndrome, Fasciotomy, Hoffmann’s syndrome, Hypothyroidism

## Abstract

**Background:**

Hoffmann’s syndrome is a rare form of hypothyroid myopathy. Only a few cases of fasciotomy in this setting have previously been reported.

**Case presentation:**

A 41-year-old Caucasian man under treatment for hypothyroidism presented with acute-onset severe pain in his forearm for no obvious reason and was admitted to our emergency room. He eventually developed compartment syndrome which necessitated surgical decompression. Soon after surgery he complained of similar symptoms in his calves. By the time his hypothyroid status was confirmed, conservative treatment and orally administered levothyroxine gradually made the pain from his calves disappear, without further surgical treatment.

**Conclusion:**

Hoffmann’s syndrome may precipitate a compartment syndrome in the absence of trauma.

## Introduction

An acute compartment syndrome of the forearm usually occurs as a result of trauma or ischemia. The non-compliant fascia surrounding the muscles essentially creates a closed compartment, which is vulnerable to all processes that expand muscle volume and therefore the intra-compartment pressure. Any pressure increase beyond a certain threshold is likely to compromise micro-circulation, and may elicit the self-perpetuating process that increases the intra-compartment pressure at the expense of circulation. The result is muscular ischemia, which produces the cardinal symptom of acute compartment syndrome; that is, excessive pain. An acute compartment syndrome represents an emergency situation, in which failure to surgically open the fascia in due time, may lead to muscle necrosis or even amputation. We present a case in which a patient developed a compartment syndrome of his forearm for no obvious reason. To the best of our knowledge, this is the first report of a patient with Hoffmann’s syndrome presenting with a compartment syndrome of the forearm described in the medical literature.

## Case presentation

A 41-year-old Caucasian man presented to hospital with intense pain in his right forearm. The pain exhibited a gradual onset over 2 days, but was abruptly worsened following the simple task of tightening a screw with a screwdriver. His background was from a middle class family with no known risk of hereditary disease. He was in a stable relationship and the father of two children, both in their twenties, from a previous marriage. After completing high school he had earned a university degree in education of children with disabilities, which was also his current profession. His alcohol consumption was moderate and he did not smoke tobacco. Despite his relatively young age, his medical record contained a comprehensive list of previous diseases. At age 25 he underwent fundoplication because of gastric reflux. The procedure was repeated 3 years later due to persisting symptoms. At 29 he was diagnosed as having Hodgkin’s lymphoma. He initially received combination chemotherapy of doxorubicin, bleomycin, vinblastine, and dacarbazine, but this was terminated due to development of leg weakness. To compensate for chemotherapy intolerance, he subsequently received radiotherapy toward lymph nodes in his neck with a total of 40 gray. Consequently, he developed hypothyroidism as a side effect.

He had previously been on thyroid hormone replacement therapy for 7 years. During these years he had been changing the thyroxine medication from synthetic levothyroxine to a preparation derived from porcine thyroid glands, before ending up with a combination of the two. The previous 6 months he had expressed frustration over a general lack of well-being, claiming that the current thyroid hormone replacement therapy did not improve his symptoms. During several visits to his endocrinologist he had communicated a strong desire to discontinue thyroxine hormone replacement therapy completely, in order to see whether it would make him feel better. The idea was supported by his endocrinologist, provided that he, the patient, would be willing to control thyroid function every week, in collaboration with his general physician. In the months leading up to the decision to abandon levothyroxine therapy completely, our patient’s medical record indicated that he was on a natural preparation derived from porcine thyroid glands corresponding to a daily dose of 19 μg of levothyroxine and 4.5 μg of liothyronine in addition to 50 μg of levothyroxine 4 days a week and 25 μg of levothyroxine the remaining 3 days. Approximately 3 months after he had in fact discontinued thyroxine hormone replacement therapy completely, he found himself being examined in the emergency unit because of acute severe pain in his right forearm. At the time of admission, his regular medication included esomeprazole 40 mg twice a day and 100 mg of ferrous sulfate once a day only.

**Fig. 1:**
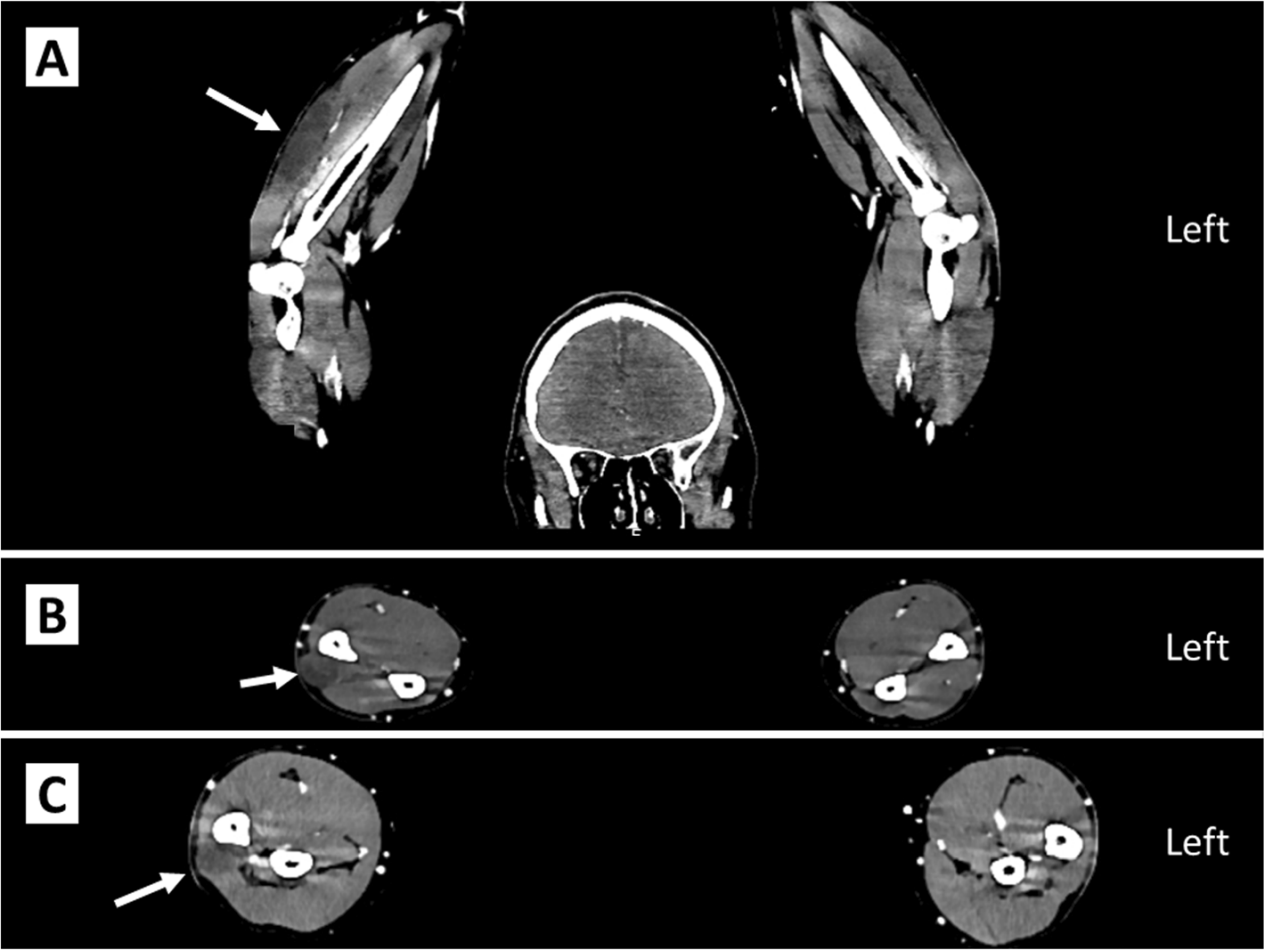
Preoperative computed tomography scan with *arrows* pointing at dark areas in the right extensor carpi ulnaris muscle, indicating edema. **a** Coronal plane; **b** transverse plane, middle; **c** transverse plane, proximal

During clinical examination in the emergency unit after midnight, severe pain was located on the dorsal side of his right forearm. His arm appeared swollen on examination, but the overlying skin was completely normal. A brief neurological examination revealed reduced sensibility to sensation on his right forearm compared to his left, especially on the ulnar side. Brachioradial reflexes were normal on both sides, whereas biceps and triceps reflexes were unsuccessfully elicited on either side. Reduced muscular power was described in his fingers and wrist on the right side, but our patient spontaneously disclosed that this was due to the pain being increased during contraction of these muscles. His blood pressure was 165/102 mmHg, pulse 90 beats/minute, respiratory rate 22 per/minute, and rectal temperature 37.0 °C. He did not present any obvious symptoms or clinical signs frequently seen in hypothyroidism, such as lethargy, hair loss, cold intolerance, or myxedema. His creatinine kinase was elevated to 1659 IU/L (range 40–280). The results of a screening of blood tests including complete blood count, liver function tests, renal function tests, and C-reactive protein were all within the reference range. The resident surgeon ordered overnight elevation of our patient’s arm and opioid analgesics. Despite repeated administration of orally administered analgesics (1 g acetaminophen every 6 hours, 50 mg tramadol hydrochloride once) and intravenously administered opioid analgesics (5 mg oxycodone every 2 hours for 8 hours, followed by 5 mg morphine every 2 hours for 8 hours), he continued to complain of intense pain in his right forearm. The next morning a clinical evaluation of our patient revealed no improvement in pain or edema. His creatinine kinase had increased slightly to 1722 IU/L, and the surgeon on call diagnosed our patient as having compartment syndrome and referred him for immediate surgery. A preoperative computed tomography (CT) scan was performed, which revealed edema in the extensor carpi ulnaris muscle (Fig. 1), but no signs of an underlying process such as bleeding, tumor, or abscess. Complementary blood tests revealed a thyroid-stimulating hormone of 30.5 μIU/ml (range 0.2–4.3) and free thyroxine of 7 μmol/L (range 9–22), indicating hypothyroidism. A summary of the blood samples and their timing is provided in Table 1. After induction of general anesthesia by a combination of remifentanil (1 μg/kg per minute), propofol (180 mg), succinylcholine (80 mg) and fentanyl (200 μg), a straightforward fasciotomy was performed. The diagnosis of compartment syndrome was subsequently confirmed by the bulging of the affected muscle following opening of the fascia.

**Table 1:** Relevant blood samples and their timing

Timing of blood samples	TSH (μIU/ml)	fT4 (μmol/L)	CK (IU/L)
Reference range	(0.20–4.30)	(9–22)	(40–280)
Four weeks before admission in EU	11.8	7	nm
Eight hours before fasciotomy	nm	nm	1722
Eight hours after fasciotomy	30.5	7	3421
Day 2 after surgery and levothyroxine therapy	24.4	6	1747
One week after discharge	1.7	13	115

*CK* creatinine kinase, *EU* emergency unit, *fT4* free thyroxine, *nm* not measured, *TSH* thyroid-stimulating hormone

The surgery successfully removed the intense pain of our patient’s forearm. However, a new problem surfaced in the postoperative ward, where he started complaining of similar intense pain in both calves, only a few hours after recovering from the forearm fasciotomy. Upon examination of his legs 3 hours after the forearm fasciotomy had been completed, his calf muscles appeared swollen, but soft and there was no clinical sign of compartment syndrome or myxedema. An ultrasound examination of his lower extremities was performed, but showed open deep veins bilaterally, excluding venous thrombosis as a source of the pain. The pain subsequently increased over the next few hours, but responded to elevation and opioid analgesics (5 mg oxycodone intravenously administered every 2 hours for 6 hours). The next morning, 18 hours after the forearm fasciotomy, the pain in his calves was still present but less severe and thyroid hormone replacement therapy was initiated with a levothyroxine dose of 50 μg a day. Over the next few days the pain in his calves gradually subsided, before disappearing completely on day 3 after the surgery. The edema of his forearm muscle also diminished over the next few days and the skin was closed on day 6 after the fasciotomy.

Our patient was followed regularly by an endocrinologist after he was discharged from hospital. His thyroid function stabilized on a substitution regime of 10 μg of liothyronine a day, in addition to 150 μg of levothyroxine 4 days a week and levothyroxine 125 μg the remaining 3 days of the week. Eight months after discharge he was seen by a neurologist. A full neurological examination only revealed normal findings, specifically demonstrating symmetrical and normal power, and normal sensation and function of his hand and wrist.

## Discussion

Hoffmann’s syndrome is a rare condition, and to the best of the authors’ knowledge it has not been previously reported in Scandinavia. Fasciotomy in this setting is even more extraordinary, with only a few published cases [1–5]. In contrast to previously published cases, the compartment that was affected in this case report was the dorsolateral compartment of the forearm, whereas the patients in previous reports all presented with compartment syndromes of tibial compartments. This is probably a reflection of the fact that anterior and lateral compartments of the lower legs are most commonly affected overall [6, 7], making this case report even more unusual.

The present case report demonstrates that the recognition of a rather clear-cut syndrome, such as acute compartment syndrome, may be obscured when the underlying pathology is misconceived. An acute compartment syndrome rarely occurs in the absence of a physical trauma or conditions which compromise limb circulation. The lack of an obvious triggering cause in the initial diagnostic workup may therefore have delayed the timing of the fasciotomy. Due to our patient’s substantial requirement for morphine analgesics overnight, and the failure of these to adequately resolve the pain in his forearm, it may be argued that the compartment syndrome should have been recognized earlier and the fasciotomy performed accordingly. The amount of skeletal muscle necrosis suffered from a compartment syndrome is directly proportional to the duration of ischemia [8], and time should not be wasted in unnecessary diagnostic workup. On the other hand, no muscle necrosis was found at the time of fasciotomy or during subsequent revisions, indicating that the surgery was, although 16 hours after admission, performed in time.

**Fig. 2:**
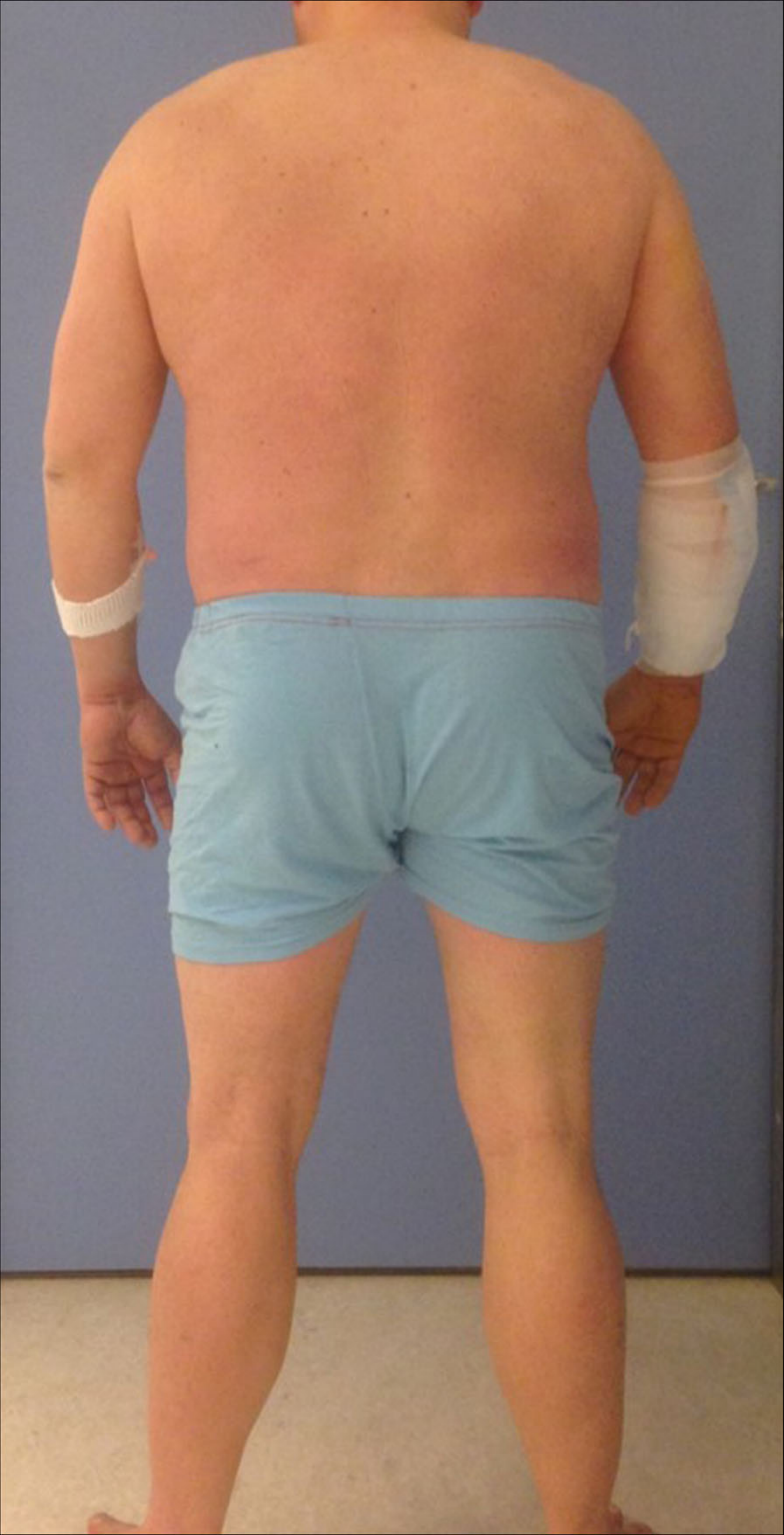
The patient photographed from the back exhibiting proximal hypertrophy of the calves, arms, and shoulders

Our patient most likely suffered from Hoffmann’s syndrome, a rare condition characterized by increased muscular mass, muscle stiffness, proximal muscle weakness, and occasional muscle cramps in the presence of hypothyroidism [9]. Although muscle stiffness and weakness were less pronounced symptoms in the present case, a more focused clinical examination revealed proximal hypertrophy of the calves, upper arms, and shoulders in a patient who denied being physically active (Fig. 2). A muscle biopsy obtained from his forearm at the time of closure of the skin only revealed acute myopathic changes with necrosis and signs of impaired circulation, which were probably caused by the compartment syndrome itself, rather than the endocrine myopathy. Another muscle biopsy obtained simultaneously from the left tensor fascia lata muscle revealed mild, nonspecific myopathic changes with increased fiber size variability and some few internalized nuclei.

The lower leg seems to be the most common location of compartment syndrome associated with hypothyroidism, with five previously published cases involving fasciotomy of tibial compartments [1–5]. To the best of our knowledge, only one patient with hypothyroid-induced compartment syndrome in the forearm has previously been reported. However, the patient in this previously reported case also presented with acute compartment syndrome of the lower extremities. The previously described bilateral compartment syndrome of the forearms appeared only the day after bilateral lower extremity fasciotomies were performed [5].

An important aspect of this case report is the use of succinylcholine during the induction of general anesthesia. Succinylcholine is a depolarizing neuromuscular agent, which through competitive inhibition of acetylcholine ultimately leads to relaxation of striated muscle. A well-known side effect of succinylcholine is its ability to induce fasciculations and postoperative myalgia [10]. The exact reason why succinylcholine causes postoperative myalgia is not clearly understood. Suggested mechanisms include muscle fiber damage produced by the shearing forces associated with these fasciculations and the release of potassium from muscle cells. However, no simple correlation between the severity of muscle fasciculations, serum potassium changes, and the development of postoperative myalgia has been proved [10]. Although the relationship between succinylcholine-induced fasciculations and postoperative myalgia remains controversial, it may seem to be a legitimate explanation in this particular case report. Due to pseudohypertrophy of this particular patient’s lower legs, his muscle-rich tibial compartments may have been more susceptible to succinylcholine-induced fasciculations, explaining the findings of painful and clinically swollen calves following the forearm fasciotomy. Muscular edema of the tibial compartments may have been further exacerbated by immobilization and fluid resuscitation.

The association between hypothyroidism and compartment syndrome was first recognized after the forearm fasciotomy and while our patient was complaining of increasing pain in both calves in the postoperative ward. As the surgical team considered surgical options for his calves as well, an endocrinologist was consulted and hypothyroidism myopathy was introduced as a possible explanation of our patient’s condition. He was treated with elevation of his legs and was given orally administered levothyroxine, after which the pain in his calves diminished within hours, before disappearing completely after a couple of days. The reason for our patient’s quick recovery from pain in his calves was probably due to the elevation of his legs and the metabolism of succinylcholine, rather than levothyroxine treatment alone. The effect of levothyroxine treatment on muscle in hypothyroid myopathy typically emerges within several weeks [11], although messenger ribonucleic acid (mRNA) synthesis in hypothyroid muscular cells has been shown to be dramatically increased within 48 hours [12]. In hindsight, it is speculated whether the use of a non-depolarizing neuromuscular blocking agent for induction of general anesthesia instead of succinylcholine would have prevented the postoperative pain in the calves experienced by our patient. In a worst case scenario, compartment syndromes may have developed in his tibial compartments as well. A similar case report involving a patient with hypothyroidism subjected to bilateral fasciotomy of the tibial compartments followed by upper extremity fasciotomies the next day has previously been reported [5], although it is not clear whether a depolarizing neuromuscular blocking agent was utilized in that particular case.

## Conclusion

Hoffmann’s syndrome may precipitate a compartment syndrome in the absence of trauma. The present case report highlights the importance of a multidisciplinary approach to complex surgical cases.

## Data Availability

Not applicable.
